# Isolation and functional validation of the *CmLOX08* promoter associated with signalling molecule and abiotic stress responses in oriental melon, *Cucumis melo* var. *makuwa* Makino

**DOI:** 10.1186/s12870-019-1678-1

**Published:** 2019-02-15

**Authors:** Chenghui Wang, Ge Gao, Songxiao Cao, Qunjie Xie, Hongyan Qi

**Affiliations:** College of Horticulture, Shenyang Agricultural University, Key Laboratory of Protected Horticulture of Education Ministry and Liaoning Province, National & Local Joint Engineering Research Center of Northern Horticultural Facilities Design & Application Technology (Liaoning), Liaoning Shenyang, 110866 People’s Republic of China

**Keywords:** Oriental melon, Lipoxygenase, Promoter, Signalling molecule, Abiotic stress

## Abstract

**Background:**

Lipoxygenases (LOXs) play significant roles in abiotic stress responses, and identification of *LOX* gene promoter function can make an important contribution to elucidating resistance mechanisms. Here, we cloned the *CmLOX08* promoter of melon (*Cucumis melo*) and identified the main promoter regions regulating transcription in response to signalling molecules and abiotic stresses.

**Results:**

The 2054-bp promoter region of *CmLOX08* from melon leaves was cloned, and bioinformatic analysis revealed that it harbours numerous *cis*-regulatory elements associated with signalling molecules and abiotic stress. Five 5′-deletion fragments obtained from the *CmLOX08* promoter—2054 (LP1), 1639 (LP2), 1284 (LP3), 1047 (LP4), and 418 bp (LP5)—were fused with a GUS reporter gene and used for tobacco transient assays. Deletion analysis revealed that in response to abscisic acid, salicylic acid, and hydrogen peroxide, the GUS activity of LP1 was significantly higher than that of the mock-treated control and LP2, indicating that the − 2054- to − 1639-bp region positively regulates expression induced by these signalling molecules. However, no deletion fragment GUS activity was induced by methyl jasmonate. In response to salt, drought, and wounding treatments, LP1, LP2, and LP4 promoted significantly higher GUS expression compared with the control. Among all deletion fragments, LP4 showed the highest GUS expression, indicating that − 1047 to − 1 bp is the major region regulating promoter activity and that the − 1047 to − 418-bp region positively regulates expression induced by salt, drought, and wounding, whereas the − 1284 to − 1047-bp region is a negative regulatory segment. Interestingly, although the GUS activity of LP1 and LP2 was not affected by temperature changes, that of LP3 was significantly induced by heat, indicating that the − 1284- to − 1-bp region is a core sequence responding to heat and the − 2054- to − 1284-bp region negatively regulates expression induced by heat. Similarly, the − 1047- to − 1-bp region is the main sequence responding to cold, whereas the − 2054- to − 1047-bp region negatively regulates expression induced by cold.

**Conclusions:**

We cloned the *CmLOX08* promoter and demonstrated that it is a signalling molecule/stress-inducible promoter. Furthermore, we identified core and positive/negative regulatory regions responding to three signalling molecules and five abiotic stresses.

**Electronic supplementary material:**

The online version of this article (10.1186/s12870-019-1678-1) contains supplementary material, which is available to authorized users.

## Background

Lipoxygenases (LOXs: EC 1.13.11.12) are a class of non-haem iron-containing dioxygenases. Plant LOXs have been classified as 9-LOX and 13-LOX, according to their different oxygen positions, and are involved in enzymatic reactions associated with the oxidation of polyunsaturated fatty acids (PUFAs) during transmutation to unsaturated fatty acid hydroperoxides [1]. Plant LOXs, which are encoded by multigene families, have been shown to play roles in the response to abiotic stresses [2–4]. Overexpression of *TomloxD* in transgenic tomatoes clearly indicated that this gene is involved in endogenous jasmonic acid (JA) synthesis, and in turn regulates the expression of plant defence genes and resistance to high temperature [5, 6]. Similarly, pepper *CaLOX1* has been shown to increase resistance to osmotic, drought, and high salinity stress [7]. Furthermore, the persimmon *DkLOX3* gene has been found to play positive roles in enhancing tolerance to salt and drought, and the stress-responsive expression of this gene in the *DkLOX3*-OX line of *Arabidopsis* was shown to be higher than that in the wild type [8]. Overexpression and silencing of the *japonica* rice *OsLOX1* gene has indicated that this gene is involved in resistance to wounding associated with JA biosynthesis [9]. *LOX* gene expression has been shown to be regulated in response to different abiotic stresses such as heat, cold, and wounding, or different signalling molecules such as methyl jasmonate (MeJA), abscisic acid (ABA), hydrogen peroxide (H_2_O_2_), and salicylic acid (SA) [10–13].

The promoter is a specific sequence of DNA upstream of the protein-coding region of a gene that contains numerous *cis*-regulatory elements and initiates transcription [14]. To date, a number of signalling molecule/stress-inducible elements have been identified in promoter regions, examples of which include a salicylic acid-responsive element (TCA) identified in tobacco [15], ABRE, a *cis*-acting element involved in ABA responsiveness, in wheat and rice [16, 17], and ethylene-responsive elements (EREs), containing an 11-bp sequence (TAAGAGCCGCC), that act as transcriptional activators or repressors of gene expression under ethylene treatment in tobacco and *Arabidopsis* [18, 19]. Heat-shock promoters have been appraised during high-temperature stress experiments in transgenic soybean and *Arabidopsis* [20, 21]. Furthermore, many core functional promoter regions have also been identified. The − 148-bp region of the grape C4C4-type RING-finger gene promoter has been demonstrated to be the core functional promoter region and plays a key role in response to heat stress [22], whereas the *CsSUS1p* promoter from *Citrus sinensis* was found to be induced in response to wounding of the phloem tissue of transgenic tobacco plants [23]. Deletion analysis of the maize type-II H^+^-pyrophosphatase gene promoter in transgenic tobacco plants has revealed that a 71-bp segment (− 219 to − 148 bp) is the key region regulating the *ZmGAPP* response to NaCl or PEG stress [24]. Furthermore, the expression levels of GUS in transgenic tobacco indicated that a 348-bp fragment of the *SbGSTU* promoter could be used for both constitutive and stress-inducible expression of genes [25]. Similarly, GUS transient assays in tobacco leaves have indicated that a 113-bp segment (− 467 to − 355 bp) from the maize phosphatidylinositol synthase gene (*ZmPIS*) is sufficient for the NaCl or PEG stress response and is considered to be the key sequence for the *ZmPIS* response to NaCl or PEG treatment [26]. In addition, stress-inducible gene expression requires the interaction between transcription factors and *cis-*acting elements in the promoter, thereby highlighting the need for functional validation of gene promoter activity in response to signalling molecules and abiotic stresses.

The oriental melon (*Cucumis melo* var. *makuwa* Makino), which has shallow roots and large thin leaves, is an important agricultural commodity and widely grown in China and other eastern Asian countries. Abiotic stresses, such as low temperature, drought, high salinity, and mechanical damage, are all unfavourable to the growth and development of melon. With the release of the entire genome sequence of melon, a new resource for the functional analysis of LOX genes in this plant has emerged [27]. To date, 18 candidate *LOX* genes have been identified in the melon genome, which have been grouped into three categories (type I 9-LOX, type I 13-LOX, and type II 13-LOX) based on phylogenetic analysis [28]. Previous studies have shown that the expression levels of melon *CmLOX08*, *CmLOX10*, *CmLOX12*, *CmLOX13*, and *CmLOX18* genes differ in response to different signal molecule and abiotic stress treatments, indicating that these five genes may play diverse functional roles in melon [29].

Phylogenetic analysis has indicated that *CmLOX08* (MELO3C011885) is a member of the type II 13-LOX genes, and is known to play an important role in biotic and abiotic stress responses [4, 28, 30, 31]. It has previously been shown that *CmLOX08* is induced in response to various stresses, including wounding, heat, and cold, or signalling molecules such as H_2_O_2_ [29]. To date, however, there have been no reports describing the mechanisms whereby the expression of *CmLOX08* is regulated in response to abiotic stress. In this study, we cloned the *CmLOX08* promoter from young leaves of the ‘Yumeiren’ cultivar of oriental melon based on sequences in melon genome databases and sought to identify putative *cis*-regulatory elements that respond to signalling molecules and abiotic stresses. To identify regions of the *CmLOX08* promoter that play a role in regulating transcription, we constructed five promoter 5′-deletion vectors using pBI121 and examined their responses in tobacco leaves subjected to a range of different signalling molecules and abiotic stresses using *Agrobacterium*-mediated transient assays. The findings of these analyses will contribute to gaining a better understanding of the molecular mechanisms underlying the response of *CmLOX08* to abiotic stress in oriental melon.

## Results

### Isolation and sequence analysis of the *CmLOX08* promoter

On the basis of the publicly available sequence in the melon genome database (http://melonomics.net), we obtained the 2054-bp 5′ flanking sequence of *CmLOX08* upstream of the ATG start codon from melon genomic DNA. Two alignments of the promoter sequence of *CmLOX08*-pro were performed based on the sequence obtained and that from the melon (*Cucumis melon* L.) genome database (*GeLOX08*-pro) using DNAMAN software. The results showed that the nucleotide sequences of *CmLOX08*-pro and *GeLOX08*-pro shared 99.61% identity. However, compared with *GeLOX08*-pro, *CmLOX08*-pro was found to have a larger number of nucleotides containing a single adenine and fewer nucleotides containing three thymines (Additional file 1).

### Analysis of *cis*-regulatory elements in the *CmLOX08* promoter

**Fig. 1:**
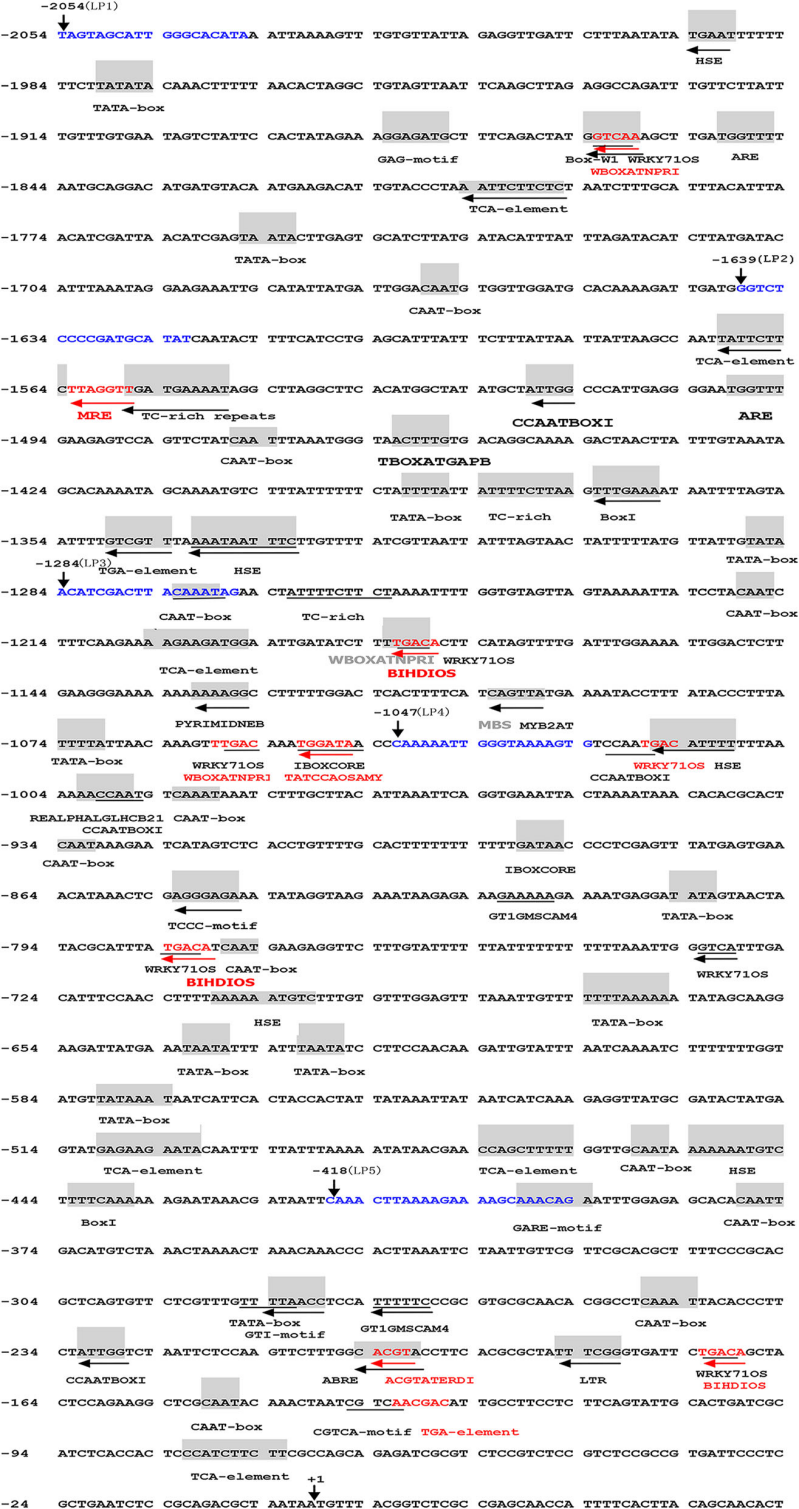
Nucleotide sequence of the *CmLOX08* promoter. The “A” of the translation initiation code “ATG” of *CmLOX08* was designated as “+ 1”. Putative *cis*-acting elements are underlined, shadowed, colored and labeled. The horizontal arrows show their directions. See Table 1 for descriptions of the elements. The vertical arrow above the sequence indicates the start point of different deletion fragments; the blue nucleotide sequences represent special primers for amplifying deletion fragments (LP1–LP5)

The *CmLOX08* promoter sequence was characterized using the online software PlantCARE and PLACE. Thirty types of potential *cis*-acting elements were detected within the 2054-bp region of the *CmLOX08* promoter (Fig. 1 and Table 1).

**Table 1:** Identification of *cis*-acting elements in the *CmLOX08* promoter using the PlantCARE and PLACE databases

*Cis*-elements	Description	Position from ATG	No.
HSE	*cis-*acting element involved in heat stress responsiveness	−1990, − 1332, − 1015, − 709, − 454	5
GAG-motif	part of a light responsive element	− 1883	1
Box-W1	fungal elicitor responsive element	− 1859	1
WRKY71OS	Core of W-box, transcriptional repressor of GA signal	− 1863, − 1182, − 1063, − 1023, − 784, − 730, − 173	7
WBOXATNPR1	Regulates NPR1, SA-induced	−1859, − 1183, − 1064	3
ARE	*cis-*acting regulatory element essential for the anaerobic induction	− 1851	1
TCA-element	*cis-*acting element involved in salicylic acid responsiveness	− 1796,-1564, − 1205, − 510, − 474, − 82	6
MRE	MYB binding site involved in light responsiveness	− 1557	1
TC-rich repeats	*cis-*acting element involved in defense and stress responsiveness	− 1548, 1384, − 1262	3
CCAATBOX1	Act cooperatively with HSE to increase the activity of the promoter	− 1515, − 1027, − 1005, − 228	4
TBOXATGAPB	Involved in light-activated transcription	− 1462	1
Box I	light responsive element	− 1367, − 443	2
TGA-element	auxin-responsive element	− 1344, − 132	2
CAAT-box	common *cis-*acting element in promoter and enhancer regions	− 1669,-1477,-1273, − 1219,-993, − 934,-778,-459,-379,-248,-150	11
BIHD1OS	Binding site of OsBIHD1 in disease resistance responses	− 1178, − 780, − 169	3
PYRIMIDINEB	involved in sugar repression and the regulation of gibberellin-responsive genes	− 1126	1
MBS	MYB binding site involved in drought-inducibility	− 1098	1
MYB2AT	Binding site for ATMYB2 that involved in the regulation of dehydration-responsive genes	−1098	1
IBOXCORE	Light-responsive element	− 1056, − 890	2
TATCCAOSAMY	Gibberellin response element in sugar sensitivity of alpha-amylase genes	− 1051	1
REALPHALGLHCB21	Required for phytochrome regulation	− 1007	1
TCCC-motif	part of a light responsive element	− 847	1
GT1GMSCAM4	Plays a role in pathogen- and salt-induced SCaM-4 gene expression	− 822, − 269	2
GARE-motif	gibberellin-responsive element	− 400	1
TATA-box	core promoter element around − 30 of transcription start	− 1980,-1756,-1391,-1288,-1074,-805,− 674,-642,-631,-580, − 286	11
GT1-motif	light responsive element	− 279	1
ABRE	*cis-*acting element involved in the abscisic acid responsiveness	−200	1
ACGTATERD1	required for etiolation-induced expression of erd1 (early responsive to dehydration)	−201	1
LTR	*cis-*acting element involved in low-temperature responsiveness	− 181	1
CGTCA-motif	*cis-*acting regulatory element involved in the MeJA-responsiveness	−136	1

Multiple core *cis*-acting elements, including 11 TATA and 11 CAAT boxes, were identified at numerous positions. Furthermore, a series of putative *cis*-regulatory elements that facilitate the inducible expression of *CmLOX08* were detected, including eight types of light-responsive elements (GAG-motif, MRE, TBOXATGAPB, Box I, IBOXCORE, REALPHALGLHCB21, TCCC-motif, and GT1-motif), nine types of hormone-responsive elements (WRKY71OS, WBOXATNPR1, TCA-element, TGA-element, PYRIMIDINEB, TATCCAOSAMY, GARE-motif, ABRE, and CGTCA-motif), two *cis*-acting elements involved in heat stress responsiveness (HSE and CCAATBOX1), a *cis*-acting element involved in low-temperature-induced expression (LTR), an element involved in pathogen- and salt-induced SCaM-4 gene expression (GT1GMSCAM4), a MYB binding site involved in drought inducibility (MBS), an ATMYB2 binding site and an element related to dehydration-responsive genes (MYB2AT and ACGTATERD1, respectively), an enhancer-like element involved in anaerobic inducibility (ARE), a fungal-inducible element (Box-W1), a binding site of OsBIHD1 involved in disease resistance responses, and three TC-rich repeats involved in defence and stress responsiveness.

### Activities of the *CmLOX08* promoter in response to signalling molecule treatments

**Fig. 2:**
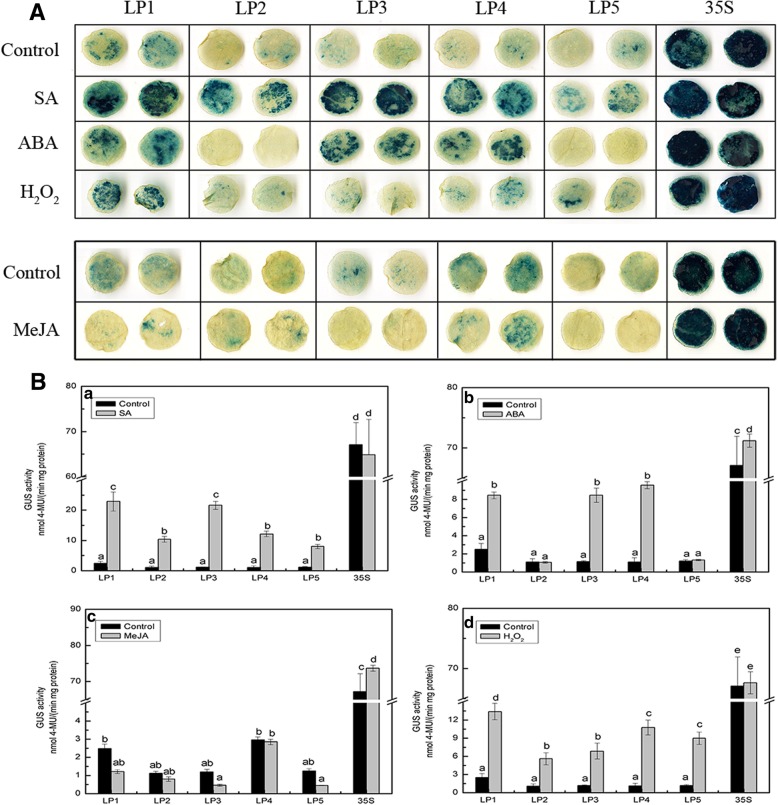
Analysis of different *CmLOX08* promoter deletion constructs in tobacco plants under signaling molecules treatments. **A** GUS histochemical staining of five deletions constructs under SA, ABA, H_2_O_2_, and MeJA treatments. **B** GUS activity of different deletion constructs under SA **(a)**, ABA **(b)**, MeJA **(c)**, and H_2_O_2_ **(d)** treatments. Values represent the means ± SD from three repeats. Different lowercase letters above the bars indicate significant differences at *P* < 0.05

To determine the role of putative *cis*-regulatory elements in the response of the *CmLOX08* promoter to signalling molecules, tobacco leaves infiltrated with *Agrobacterium* harbouring *CmLOX08* promoter fragments of five different deletion lengths were treated with SA, ABA, MeJA, and H_2_O_2_, followed by GUS histochemical staining and fluorometric assay analyses (Fig. 2).

In response to treatment with SA, each of the five deletion structure was intensely stained and the GUS activity of the LP1–LP5 promoter fragments was significantly increased by 9.18-, 9.48-, 18.65-, 10.99-, and 6.66-fold, respectively, compared with the mock-treated control after treatment with SA (Fig. 2A, B, a). These results indicate that at least one SA-responsive element is located in the promoter region from − 418 to − 1, although additional *cis*-elements could be present in regions further upstream. After treatment with ABA, the LP1, LP3, and LP4 promoter constructs were strongly stained compared with the control, whereas the LP2 and LP5 constructs remained unstained (Fig. 2A). We observed that GUS activity increased significantly for the LP1, LP3, and LP4 fragments, whereas no significant change was detected for LP2 and LP5 (Fig. 2B, b). These results indicate that the promoter regions from − 2054 to − 1639 and − 1284 to − 418 play a positive role in the response to ABA and may contain the *cis*-element that responds positively to ABA treatment. Furthermore, we found that the promoter region from − 1639 to − 1284 may contain repressor elements that respond negatively to ABA treatment. In response to treatment with MeJA, we were unable to detect any significant staining or GUS activity in any of the *CmLOX08* promoter deletion fragments in comparison with the control treatment (Fig. 2A, B, c). For H_2_O_2_ treatment, the changes in GUS activity were consistent with those observed following SA treatment (Fig. 2B, d). Accordingly, these findings indicate that, whereas the *CmLOX08* promoter responds positively to ABA, SA, and H_2_O_2_, it shows no detectable response to MeJA.

### Analysis of abiotic stress-induced activity of the *CmLOX08* promoter

To examine the activity of the *CmLOX08* promoter in response to environmental stress and to identify the corresponding *cis*-regulatory regions, tobacco plants infiltrated with *Agrobacterium* harbouring *CmLOX08* promoter fragments of five different deletion lengths were subjected to salt, drought, wounding, heat, and cold stresses.

**Fig. 3:**
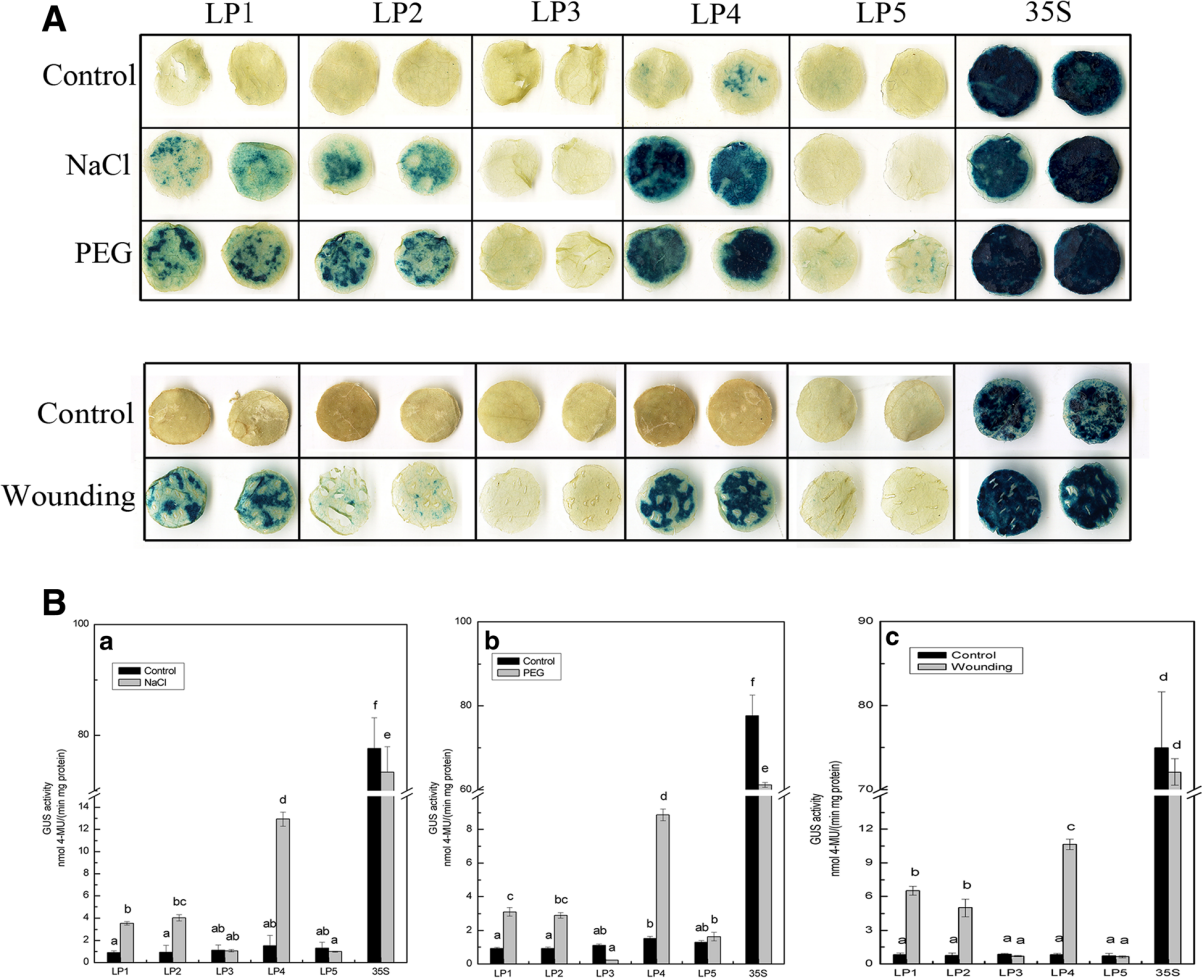
Analysis of *CmLOX08* promoter deletion constructs in tobacco plants under salt, drought and wounding treatments. **A** GUS histochemical staining of five deletions constructs in tobacco plants under 200 mM NaCl, 18% (*w*/*v*) PEG 6000 and wounding treatments. **B** GUS activity of different deletion constructs in tobacco plants under 200 mM NaCl **(a)**, 18% (w/v) PEG 6000PEG **(b)** and wounding **(c)** treatments. Values represent the means ± SD from three repeats

For NaCl and PEG treatments, the promoter activities of LP1–LP5 were examined in leaves by incubating the detached leaves in half-strength liquid MS medium supplemented with 200 mM NaCl (salt stress treatment) or 18% PEG 6000 (drought stress treatment). pBI121 (35S) (positive control) and p121GUS (negative control) plants were also treated in parallel. In response to osmotic stress (NaCl or PEG) treatments, there was a significant increase in the inducible GUS activity of leaves harbouring the LP1, LP2, and LP4 deletion fragments, whereas no significant changes were detected for the LP3 and LP5 fragments when compared with the untreated controls (Fig. 3B, a and b). Furthermore, we observed that induced GUS activity was highest in the LP4 fragment (the promoter region from − 1047 to − 1), indicating that this region contains elements of a salt- or drought-inducible nature and may promote high levels of gene expression. In response to wounding treatment, the GUS activity induced by LP1, LP2, and LP4 fragments increased by 7.74-, 6.69-, and 12.77-fold, respectively, compared with the control, whereas that induced by LP3 and LP5 remained stable (Fig. 3B, c). These results indicate that there are no wounding-responsive-elements present in the − 418 to − 1 region of the *CmLOX08* promoter, whereas in contrast, the region from − 1047 to − 418 contains major *cis* elements that respond to wounding treatment.

**Fig. 4:**
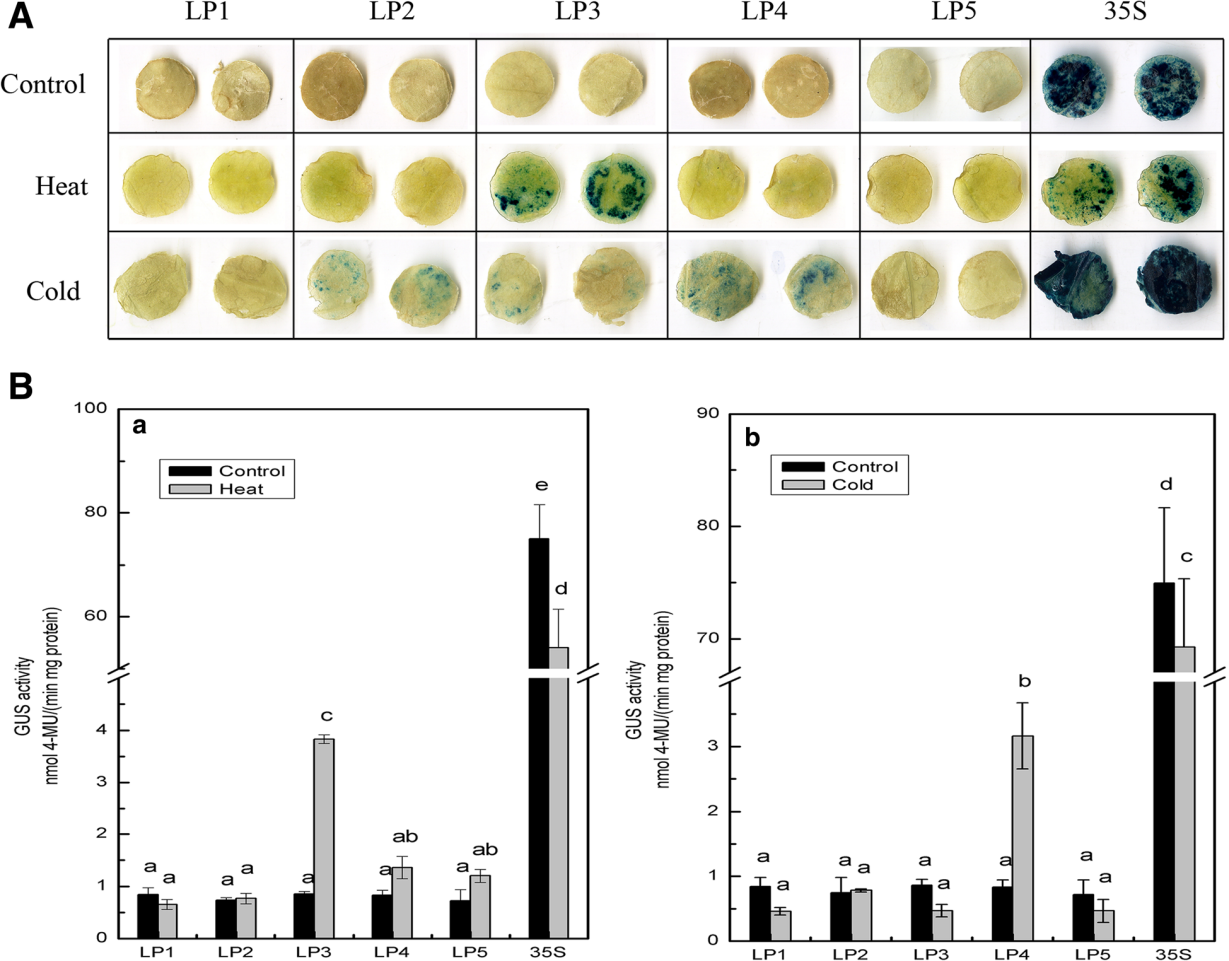
Analysis of different *CmLOX08* promoter deletion constructs under heat or cold treatments. **A** GUS histochemical staining of five deletions constructs under heat and cold treatments. **B** GUS activity of different deletion constructs under heat **(a)** and cold **(b)** treatments. Values represent the means ± SD from three repeats

When tobacco leaves were exposed to high temperature treatment, the highest level of GUS activity was detected in the LP3 deletion structure, which increased significantly (4.46-fold) compared with that of the control treatment (Fig. 4B, a). However, no significant changes in GUS activity were detected for any of the other deletion fragments, thereby indicating that the key *cis* elements that respond to heat reside in the − 1284 to − 1047 region of the promoter, and that repressor elements may exist in the − 2054- to − 1284-bp region of the *CmLOX08* promoter. The results of GUS activity assays under cold treatment were essentially similar to those obtained for heat treatment. Furthermore, the GUS activity of the LP4 deletion structure was 3.79-fold higher than that of the mock-treated control, whereas the other deletion structures showed no significant change compared with the control (Fig. 4B, b). Accordingly, these results indicate that the promoter region from − 1047 to − 418 harbours a cold-inducible *cis*-element and that repressor elements may be present in the − 2054- to − 1047-bp fragment of the *CmLOX08* promoter.

In addition to the aforementioned treatments, we also examined GUS activity in positive control tobacco leaves infected with *Agrobacterium* harbouring p121GUS:35S, which showed strong GUS activity (Figs. 2, 3 and 4), whereas negative control tobacco leaves infected with *Agrobacterium* harbouring p121GUS showed no GUS activity (Additional file 2).

## Discussion

In plants, LOX enzymes are involved in different forms of stress response, including that induced by wounding or different signalling molecules such as MeJA and SA, which are well-known modulators of defence responses [32, 33]. To investigate how *CmLOX08* gene expression could be regulated when oriental melon is subjected to different abiotic stresses, we initially cloned the 2054-bp promoter of *CmLOX08* and identified therein several *cis*-regulatory elements that are predicted to respond to signalling molecules and environmental stresses, based on reference to the PlantCARE and PLACE databases (Fig. 1 and Table 1). Subsequently, we performed deletion analysis of the *CmLOX08* promoter, with the aim of determining the major promoter regions that mediate the responses to signalling molecules and abiotic stresses using an *Agrobacterium*-mediated transient assay in tobacco leaves.

Our data showed that the GUS activity of all the examined *CmLOX08* promoter deletion structures was significantly induced after SA treatment (Fig. 2B, a). Furthermore, we found that, at numerous positions, the *CmLOX08* promoter contains a TCA-element, which is a *cis*-acting element involved in SA responsiveness [15]. Thus, the TCA motif may play a role in regulating the expression of *CmLOX08* that is similar to that observed in *GPP* from *Actinidia deliciosa* when SA acts as a signal molecule [34]. We observed that the expression level of *CmLOX08* was significantly reduced after SA treatment [29], which may due to associated transcription factors that play a negative regulatory role with respect to *CmLOX08* transcription in response to SA treatment [35, 36].

In plants, ABA is a broad-spectrum phytohormone involved in integrating various stress signal transduction pathways during the response to abiotic stresses [37]. The signalling pathways involved in the response to abiotic stress are mainly divided into ABA-dependent and ABA-independent signalling pathways [38–40]. These pathways can be regulated by ABREs (abscisic acid-responsive elements), DRE/CRTs (dehydration-responsive element/C repeats), or MYB and MYC recognition motifs [41, 42]. Although we detected the presence of ABRE, MYB2AT, and multiple MYB-like (T/AGTTA/T) elements spread across the entire region of the *CmLOX08* promoter, we were unable to locate any DRE/CRTs elements (Fig. 1). Furthermore, we also found that in response to ABA treatment, the GUS expression induced by the promoter deletion fragments LP1, LP3, and LP4 was significantly higher than that in the mock-treated control (Fig. 2B, b). On the basis of these observations, we can infer that the ABRE- and MYB-binding sites in the promoter of *CmLOX08* may play a significant role in the response to exogenous ABA. Surprisingly, ABA treatment had no effect on the gene expression of *CmLOX08* [29], and hence, further studies are needed to determine whether *CmLOX08* responds to abiotic stress via an ABA-independent pathway.

Previous studies have shown that ABA, SA, MeJA, and H_2_O_2_ interact with each other under stress conditions [12]. For example, in tomato, ABA and MeJA were shown to synergistically promote expression of the *PIN2* gene [43], whereas in the present study, unlike the response to ABA, GUS activity of the *CmLOX08* promoter was not induced by MeJA (Fig. 2B, c), indicating that there may be no interaction between ABA and MeJA in the regulation of *CmLOX08* gene expression. Interestingly, the expression of *CmLOX08* was significantly down-regulated after 3 h under MeJA treatment [29]. This response may be attributable to other factors that affect the expression of *CmLOX08* independently of MeJA treatment.

In contrast, we found that treatment with H_2_O_2_ resulted in a significant increase in GUS activity of the *CmLOX08* promoter (Fig. 2B, d), which is consistent with the up-regulation of *CmLOX08* [29]. In this regard, it is worth noting that the *CmLOX08* promoter has no H_2_O_2_-inducible motifs (Fig. 1). These results indicate that certain *cis*-acting regulatory elements involved in H_2_O_2_ responsiveness may be present in the *CmLOX08* promoter. However, the sequences of H_2_O_2_-inducible motifs need to be further confirmed.

In our previous studies, we found that NaCl and PEG treatments could promote an up-regulation of *CmLOX08* expression levels (Additional file 3). The transient expression results obtained in the present study revealed that the GUS activities of promoter deletion fragment LP4 were the highest compared with those of other deletion fragments under NaCl and PEG treatments (Fig. 3B, a and b), which indicates that the 1047-bp (LP4) segment may contain *cis*-acting elements that are induced by the aforementioned abiotic stresses. Interestingly, we noted the presence of a GT1GMSCAM4 motif in LP4 (Fig. 1), which is a *cis*-acting element involved in salt responsiveness [44]. Therefore, collectively, these results indicate that the GT1GMSCAM4 motif may play a role in regulating the expression of *CmLOX08* in response to salt stress. In contrast, we were unable to detect any drought-inducible elements in the 1047-bp (LP4) segment, indicating that this region may contain a hitherto uncharacterized element that is crucial for drought responsiveness. In our wounding treatment, GUS activity was induced by the LP1, LP2, and LP4 promoter fragments (Fig. 3B, c), with expression being significantly increased at 1.5, 3, 6, and 12 h after wounding [29]. We noted, however, that there are no recognized associated response elements in the *CmLOX08* promoter. We did, nevertheless, detect a GARE motif in the 1047-bp (LP4) segment (Fig. 1), and although this motif has been recognized as a gibberellin-responsive element in the promoter region of *SFR2* in *Brassica oleracea*, it may play an important role in the response to wounding [45]. Thus, these results tend to indicate that the promoter activity induced by mechanical damage is similar to that induced in response to exogenous stimuli [34, 40, 46]. Currently, however, we still have very limited knowledge regarding the complex interactions between promoters and transcription factors at the transcriptional level [47]. Accordingly, in order to further elucidate the regulatory mechanism of *CmLOX08* in response to abiotic stress, it will be particularly important to study the transcription factors that combine with the *CmLOX08* promoter under different abiotic stress treatments.

With the exception of LP3, for which GUS activity was significantly increased, we were unable to detect any GUS expression induced by the *CmLOX08* promoter deletion fragments when we subjected tobacco plants to heat stress (Fig. 4B, a). Within the 1047-bp (LP4) region, there are six *cis*-acting elements involved in heat stress responsiveness (three HSEs and three CCAATBOX1s), which can bind heat stress transcription factors [48]. However, we unable to detect any heat-inducible elements in the 237-bp region between LP3 and LP4 (Fig. 1). Our observation that the GUS activity of LP3 was higher than that of LP4 can probably be attributed to the fact that the 237-bp region between LP3 and LP4 contains enhanced elements involved in defence and stress responsiveness (TC-rich repeats) or contains uncharacterized heat-inducible *cis*-acting elements [49]. Given that *CmLOX08* expression is also significantly induced at various time points after heat treatment [29], we infer that HSE and CCAATBOX1 are the main heat-responsive elements and that TC-rich repeats also play an important role in the response to heat stress.

Low temperature was found to increase the GUS activity of the LP4 deletion structure (Fig. 4B, b), which may be attributable to the presence of an LTR element that is responsive to low temperature [50]. Interestingly, low temperature significantly increased the expression of *CmLOX08* after 6 h under cold stress [29]. We thus speculate that the LTR motif may play a role in regulating the expression of *CmLOX08* in response to cold stress.

## Conclusion

In this study, we cloned the promoter region of the oriental melon *CmLOX08* gene and subsequently sought to identify putative *cis*-regulatory elements that respond to signalling molecules and abiotic stresses by reference to the PlantCARE and PLACE databases. The results of GUS histochemical staining and fluorescence assays indicated that activity of the *CmLOX08* promoter is regulated by various signalling molecules and abiotic stresses, and that the promoter generally functions as a signalling molecule/stress-inducible promoter. By analysing the differing responses of *CmLOX08* promoter deletion fragments of various lengths to signalling molecules and abiotic stresses, we were able to characterize the core and positive and negative regulatory regions that show responsiveness to three different signalling molecules and five types of abiotic stress, respectively. The data generated in this study will enrich the existing inducible promoter resources and provide useful information for further study of the mechanisms whereby *CmLOX08* is regulated when oriental melon is subjected to different abiotic stresses.

## Methods

### Plant materials, growth conditions, and bacterial strains

Oriental melons (*Cucumis melo* var. *makuwa* Makino) cultivar ‘YuMeiren’, from the Yijianpu Mishijie Melon Research Institution, Changchun, China, were grown individually in a culture room at 25 ± 2 °C, under a 14-h light/10-h dark photoperiod at Shenyang Agricultural University, Shenyang, China. The young leaves of 1-month-old seedlings were harvested, immediately frozen in liquid nitrogen, and stored at − 80 °C until used for cloning of the *CmLOX08* promoter. Tobacco (*Nicotiana benthamiana*) preserved in our laboratory was raised for 6 weeks in a mix of peat, perlite, and vermiculite (2:1:1, *v*/v/v) at 25 °C under a 16/8 h day/night cycle followed by using for agro-infiltration. *Escherichia coli* strain DH5α (Tiangen Biotech, China) was used for the cloning and propagation of all recombinant plasmid vectors, and *Agrobacterium tumefaciens* strain GV3101 (Weidi Biotech, China) was used for tobacco leaf infiltration.

### Isolation of the *CmLOX08* promoter

Genomic DNA was isolated from young melon leaves using a NuClean Plant Genomic DNA Kit (Kangwei Biotech, China) following the manufacturer’s protocol and used for cloning of the *CmLOX08* promoter. On the basis of the *CmLOX08* promoter sequence obtained from the melon genome database (http://melonomics.net, accession number: MELO3C011885), we designed a pair of primers, LOX08pro-F and LOX08pro-R (Table 2), which were used to amplify the full-length genomic sequence using melon genomic DNA as a template. *CmLOX08* promoter fragments were amplified using high-fidelity PrimeSTAR™ HS DNA polymerase (Takara, Japan) in a 50-μL reaction mix containing 2 μL genomic DNA and 0.2 μM concentrations of each primer. The PCR amplification conditions were as follow: 30 cycles of 10 s at 98 °C, 15 s at 54 °C, and 2.5 min at 72 °C, with a final extension at 72 °C for 10 min. The amplified fragments of approximately 2 kb in size were purified using a MiniBEST Agarose Gel DNA Extraction Kit (Takara, Japan), according to the manufacturer’s protocols, and inserted via TA-cloning into the pMD18-T vector (Takara, Japan) following the addition of poly A tails (Additional file 4). Plasmids containing inserts of the expected size, identified by plasmid PCR, were sequenced by Invitrogen (Shanghai, China). Finally, we obtained a 2054-bp fragment upstream of the translation start codon of *CmLOX08*, which was considered to be the full-length promoter and was designated pLOX08-pro.

**Table 2:** PCR primers used in the current study

Primer name	Primer sequence (5′–3′)
LOX08pro-F	TAGTAGCATTGGGCACATA
LOX08pro-R	TATTAGCGTCTGCGGAGA
LP1-F1	AGCTT TAGTAGCATTGGGCACATA
LP1-F2	T TAGTAGCATTGGGCACATA
LP2-F1	AGCTT GGTCTCCCCGATGCATAT
LP2-F2	T GGTCTCCCCGATGCATAT
LP3-F1	AGCTT GTATAACATCGACTTACAAATAG
LP3-F2	T GTATAACATCGACTTACAAATAG
LP4-F1	AGCTT CAAAAATTGGGTAAAAGTG
LP4-F2	T CAAAAATTGGGTAAAAGTG
LP5-F1	AGCTT CAAACTTAAAAGAAAAGCAAACAG
LP5-F2	T CAAACTTAAAAGAA AAGCAAACAG
LP-R1	C TATTAGCGTCTGCGGAGA
LP-R2	GATCC TATTAGCGTCTGCGGAGA

Nucleotides underlined were used for producing *Hin*dIII and *Bam*HI restriction site

### Analysis of *cis*-regulatory elements in the *CmLOX08* promoter

On the basis of the cloned and sequenced fragments, sequence analysis of the *cis-*regulatory elements of the *CmLOX08* promoter was performed using PlantCARE (http://bioinformatics.psb.ugent.be/webtools/plantcare/html/) and PLACE (http://www.dna.affrc.go.jp/PLACE/) databases [51, 52].

#### Construction of *CmLOX08* promoter::GUS plasmids

For functional validation of the *CmLOX08* promoter, five 5′-deletion fragments encompassing different lengths of the *CmLOX08* promoter (− 2054 bp, − 1639 bp, − 1284 bp, − 1047 bp, and − 418 bp to − 1 bp), and containing *Hin*dIII and *Bam*HI restriction sites, were amplified using five sets of specific PCR primers (Table 2) and two PCR reactions according to a method described in the literature [53]. Using this method, the fact that the inserted fragment might have the same restriction site as the vector used in the subsequent step was not a relevant consideration. Using the pLOX08-pro plasmid as template DNA, the 2054-bp *CmLOX08* promoter fragments containing either *Hin*dIII or *Bam*HI restriction sites were amplified using the first (LP1-F1 and LP-R1) and second (LP1-F2 and LP-R2) pair of primers, respectively, to eventually generate two different PCR fragments. Similarly, two different PCR fragments for the other four deletion fragments of the *CmLOX08* promoter (1639, 1284, 1047, and 418 bp) were acquired using the same primer pairs (Table 2). PCR reactions were performed with high-fidelity PrimeSTAR™ HS DNA Polymerase (Takara, Japan) according to the manufacturer’s instructions, using the same PCR conditions as described above. The PCR products were purified using a MiniBEST Agarose Gel DNA Extraction Kit (Takara, Japan) according to the manufacturer’s protocols.

**Fig. 5:**
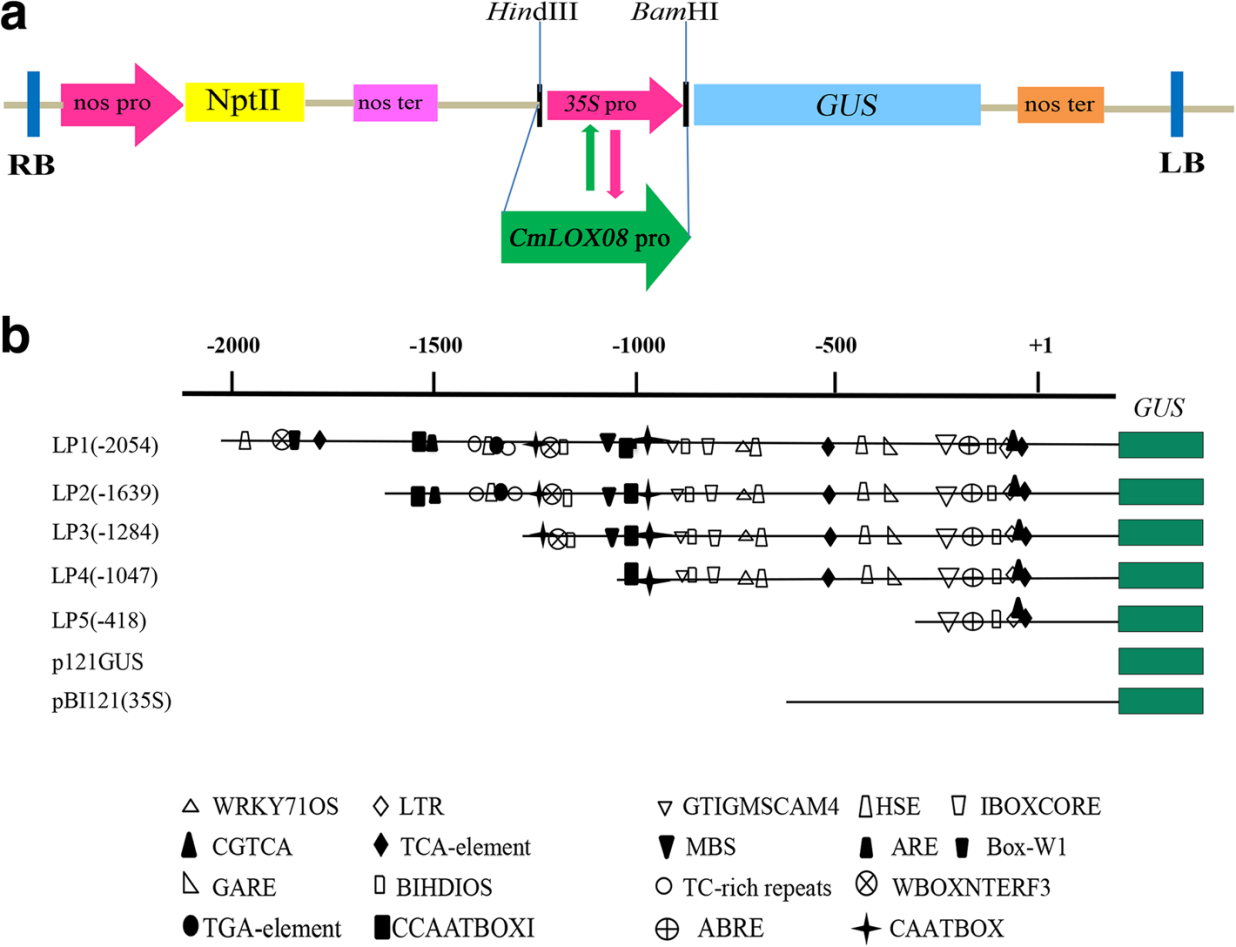
Transient transformation of tobacco plants with *CmLOX08* promoter::GUS constructs. **a** Schematic representation of *CmLOX08* promoter::GUS vector constructs. NPT II, neomycin phosphotransferase II gene; nos ter, nopaline synthase terminator; GUS, β- glucosidase gene. RB and LB, left and right T-DNA borders. The insertion position of the CmLOX08 promoter in the vector is indicated with restriction enzyme sites (*Hin*dIII and *Bam*HI). **b** Schematic representation of the different 5’deletion *CmLOX08* promoter constructs used to assay GUS activity in tobacco leaves. These constructs are based on the pBI121vector. The main *cis*-elements are represented with different patterns

Similar amounts of the two different purified PCR fragments of the *CmLOX08* promoter, acquired as described above, were added to a 50-μL reaction mixture containing 1 μL T4 polynucleotide kinase (Takara, Japan) and 1 μL 100 mM ATP (Takara, Japan). PCR was performed using the following reaction conditions: 90 min at 37 °C, 5 min at 95 °C, and 10 min at 25 °C. Mixtures containing DNA fragments of the *CmLOX08* promoter were subsequently ligated into the pBI121 binary expression vector digested with *Hin*dIII and *Bam*HI (Takara, Japan) using T4 DNA ligase (Takara, Japan) to generate the recombinant vector designated LP1, in which the *CaMV35S* promoter of vector pBI121 was replaced with the 2054-bp *CmLOX08* promoter fragment. Recombinant vectors containing the other four promoter deletion fragments were constructed using the same method and designated LP2 (1639 bp), LP3 (1284 bp), LP4 (1047 bp), and LP5 (418 bp), respectively (Fig. 5). The five different recombinant vectors were verified by plasmid PCR (Additional file 5) and sequenced prior to being transformed into *Agrobacterium tumefaciens* strain GV3101 using the freeze–thaw transformation technique. In addition, the pBI121 (p121GUS:35S) vector was used as positive control and p121GUS was used as a negative control. The recombinant vector p121GUS was generated from the pBI121 vector by digesting with *Hin*dIII and *Bam*HI (Takara, Japan) to excise the *CaMV 35S* promoter, followed by filling the 5′ overhangs of the linearized vector with T4 DNA polymerase (Takara, Japan) and ligating the blunt ends using T4 DNA ligase (Takara, Japan) according to the manufacturer’s instructions.

#### *Agrobacterium*-mediated transient expression assay

*Agrobacterium*-mediated transient expression assays were performed as described previously [54, 55]. A single colony of *Agrobacterium* strain GV3101 harbouring one of the five different recombinant binary vectors was inoculated into 2 mL YEB medium supplemented with 50 μg mL^− 1^ rifampicin and 50 μg mL^− 1^ kanamycin and grown at 28 °C and 200 rpm for 24 h. Thereafter, 1 mL of the resulting culture was transferred into 50 mL YEB medium containing 50 μg mL^− 1^ rifampicin, 50 μg mL^− 1^ kanamycin, 10 mM ethanesulfonic acid (pH 5.7 MES), and 20 μM acetosyringone (AS) and grown at 28 °C and 200 rpm for 24 h. The *Agrobacterium* cells were harvested by centrifugation at 5000 rpm for 10 min at room temperature, and following the removal of supernatant, the pelleted cells were resuspended in infiltration medium (10 mM pH 5.7 MES, 10 mM MgCl_2_, and 150 μM AS). The cell suspension was diluted to OD_600_ = 0.8 using infiltration medium, and then incubated at room temperature for 3 h in the dark. The *Agrobacterium* suspensions harbouring recombinant binary vectors were then injected into the abaxial surfaces of the leaves of 6-week-old tobacco plants using a 1-mL needleless syringe. The inoculated tobacco plants were subsequently maintained in a growth cabinet under a 16/8 h day/night cycle at 25 °C for 48 h.

#### Signalling molecule and abiotic stress treatments

For analysis of the *cis*-regulatory element of the *CmLOX08* promoter, agro-infiltrated tobacco plants were treated with SA, ABA, MeJA, and H_2_O_2_ to characterize promoter induction in response to signalling molecule treatments. For SA, ABA, and H_2_O_2_ treatments, the tobacco plants were sprayed with 1 mM SA, 0.1 mM ABA, or 10 mM H_2_O_2_ in distilled water, respectively, whereas control plants were sprayed with distilled water. For MeJA treatment, the tobacco plants were sprayed with 0.1 mM MeJA dissolved in 10% ethanol and control plants were sprayed with 10% ethanol.

We also exposed the agro-infiltrated tobacco plants to a variety of abiotic stresses, namely, low and high temperatures, mechanical wounding, salinity, and drought, to characterize the extent to which promoter activation occurs in response to these stresses. For the low and high temperature treatments, the plants were maintained in a growth cabinet at 4 °C or 42 °C, respectively. For the wounding treatment, the tobacco leaves (an area of approx. 20 cm^2^) were pricked 200 times with the needle of a 10-mL syringe. Control tobacco plants were placed in a growth cabinet at 25 °C without any treatment.

For salinity and drought stress treatments, we followed the methods described by Hou et al. [24]. Leaf discs obtained from infiltrated plants were floated on half-strength liquid MS medium supplemented with either 200 mM NaCl (salt stress treatment) or 18% (*w*/*v*) PEG 6000 (drought stress treatment) for 24 h. Infiltrated leaves incubated in half-strength liquid MS medium were considered a control.

The leaves of the tobacco plants subjected to signalling molecule and abiotic stress treatments and their controls were used for GUS analysis after 24 h. All experiments were repeated three times.

#### Histochemical staining and fluorometric assays for detecting GUS activity

Histochemical staining was performed in accordance with the procedures described by Jefferson [56]. Tobacco leaves were punched with a hole punch to obtain leaf discs with a diameter of 1 cm and these were incubated in GUS staining solution [50 mM sodium phosphate buffer (pH 7.0), 0.5 mM potassium ferricyanide, 0.5 mM potassium ferrocyanide, 10 mM EDTA, 1 mM X-Gluc (Sangon, Shanghai, China), and 0.1% Triton X-100] at 37 °C for 24 h. After staining, the tissues were bleached with 70% ethanol and photographed using a scanner.

Transient expression of GUS activity in the treated tobacco leaves was measured as described previously [57]. Tobacco leaf tissue (0.15 g) was homogenized in an extraction buffer [50 mM sodium phosphate buffer (pH 7.0), 10 mM EDTA (pH 8.0), 0.1% Triton X-100, 0.1% (w/v) sodium dodecylsulfate, and 0.1% β-mercaptoethanol] at 4 °C, and centrifuged at 12,000×*g* for 15 min at 4 °C. Aliquots (100 μL) of the resulting supernatant were mixed with 600 μL GUS assay solution [1 mM methyl-4-umbelliferyl-d-glucuronide in extraction buffer (4-MUG), Sigma, USA] pre-warmed to 37 °C and incubated at 37 °C for 30 min. A portion of this mixture (100 μL) was then added to 900 μL stop buffer (0.2 M Na_2_CO_3_). Fluorescence was measured using a Cary Eclipse Fluorescence Spectrophotometer (Agilent, USA) at excitation and emission wavelengths of 365 nm and 455 nm, respectively. Stop buffer and 10 nM to 80 nM 4-methylumbelliferone (4-MU) were used for calibration and standardization. Protein concentrations were determined using a Bradford protein assay kit (Takara, Japan) following the manufacturer’s instructions with bovine serum albumin used as a standard. GUS activity is presented as nM of 4-MU generated per min per mg soluble protein. GUS measurements were repeated three times.

#### Statistical analysis

Data are expressed as the mean values ± standard deviation (SD) of three independent experiments and were analysed using SPSS statistical software (IBM SPSS statistics 18.0, Chinese version) using an independent sample *t* test. A *P* value ≤0.05 was considered significant. Charts presenting data were generated using Origin software (version 8.0).

## Additional files

Additional file 1: Alignment of the nucleotide sequences of *CmLOX08*-pro and *GeLOX08*-pro. *CmLOX08*-pro promoter: oriental melon (*Cucumis melo* var. *makuwa* Makino); *GeLOX08*-pro promoter: the melon (*Cucumis melon* L.) genome database. Identical and dissimilar nucleotides are shown on a background of blue and gray, respectively. The two primers LOX08pro-F and LOX08pro-R which cloned the *CmLOX08* promoter are indicated by arrows. The translation initiation codon (ATG) is framed and marked for “+ 1”. (PDF 498 kb)

Additional file 2: GUS histochemical staining of the p121GUS tobacco leaves as negative control. (PDF 125 kb)

Additional file 3: Expressions of *CmLOX08* at various time points under 50 mM NaCl (a) and drought (b) treatments. (PDF 148 kb)

Additional file 4: PCR amplification of *CmLOX08* full length promoter. Lane M: DL5000 DNA Marker; lane 1 to 5: *CmLOX08* full length promoter fragment. (PDF 123 kb)

Additional file 5: The five different length recombinant vectors were verified by plasmid PCR. Lane M: DL5000 DNA Marker. Lane 1 to 4: LP1 (2054 bp); lane 5 to8: LP2 (1639 bp); lane 9 to 12:LP3 (1284 bp); lane 13 to 16: LP4 (1047 bp); lane 17 to 20: LP5 (418 bp). (PDF 126 kb)

## Abbreviations

ABA: Abscisic acid
AS: Acetosyringone
GUS: β-Glucuronidase
H_2_O_2_: Hydrogen peroxide
JA: Jasmonic acid
LOX: Lipoxygenase
MeJA: Methyl jasmonate
MES: Ethanesulfonic acid
MgCl_2_: Magnesium chloride
PUFA: Polyunsaturated fatty acid
SA: Salicylic acid
