# Supplementary material for: Isolation and functional validation of the CmLOX08 promoter associated with signalling molecule and abiotic stress responses in oriental melon, Cucumis melo var. makuwa Makino
Source: BMC Plant Biol. 2019 Feb 15;19:75. doi: 10.1186/s12870-019-1678-1 (PMC6377772; doi:10.1186/s12870-019-1678-1)

Additional file 1: Alignment of the nucleotide sequences of *CmLOX08*-pro and *GeLOX08*-pro.

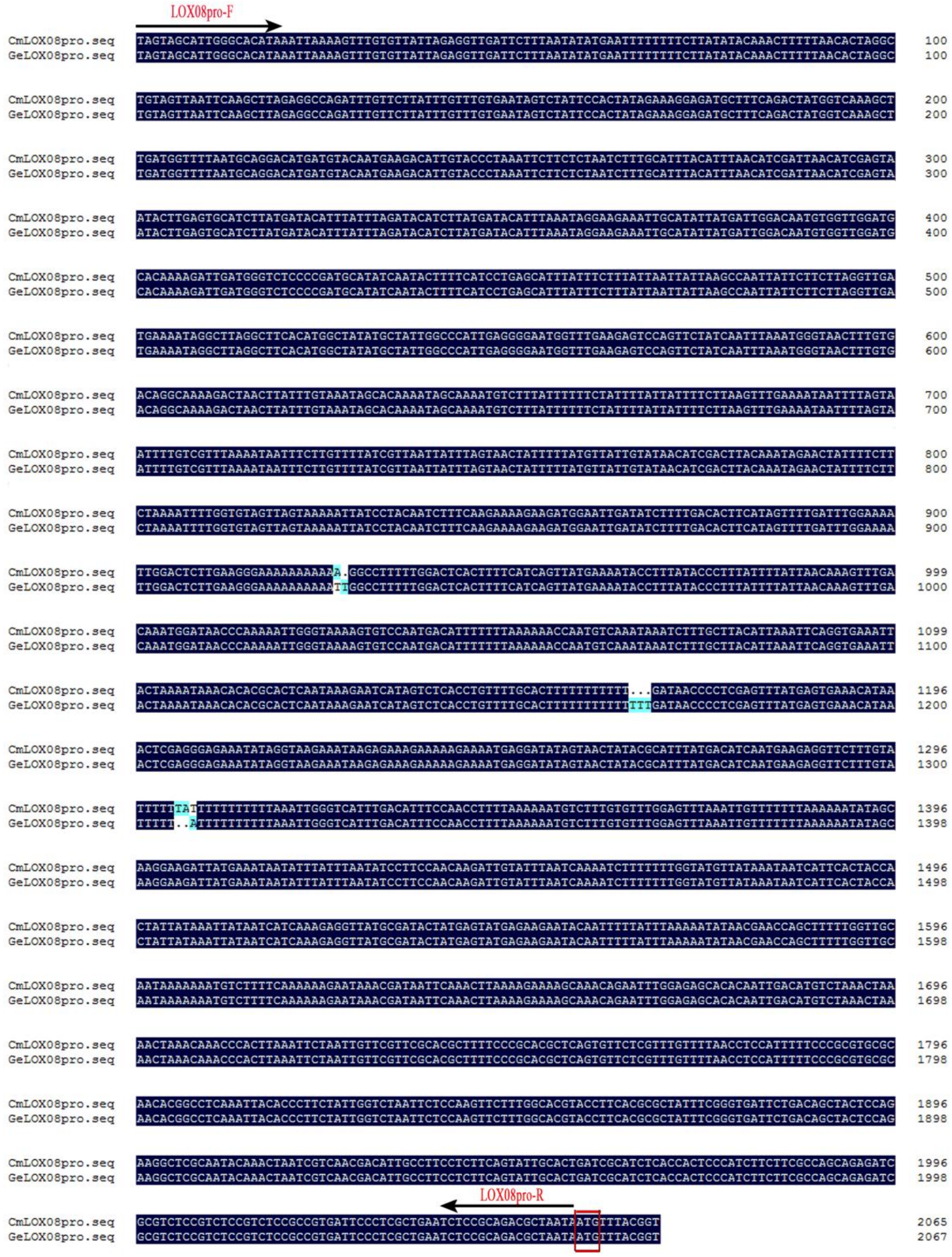

Supplement: Supplementary file 1 — Alignment of the nucleotide sequences of CmLOX08-pro and GeLOX08-pro. CmLOX08-pro promoter: oriental melon (Cucumis melo var. makuwa Makino); GeLOX08-pro promoter: the melon (Cucumis melon L.) genome database. Identical and dissimilar nucleotides are shown on a background of blue and gray, respectively. The two primers LOX08pro-F and LOX08pro-R which cloned the CmLOX08 promoter are indicated by arrows. The translation initiation codon (ATG) is framed and marked for “+ 1”. (PDF 498 kb) [file 12870_2019_1678_MOESM1_ESM.pdf]
