# Supplementary figures and images for: Isolation and functional validation of the CmLOX08 promoter associated with signalling molecule and abiotic stress responses in oriental melon, Cucumis melo var. makuwa Makino
Source: BMC Plant Biol. 2019 Feb 15;19:75. doi: 10.1186/s12870-019-1678-1 (PMC6377772; doi:10.1186/s12870-019-1678-1)

**Additional file 2:** GUS histochemical staining of the p121GUS tobacco leaves as negative control.

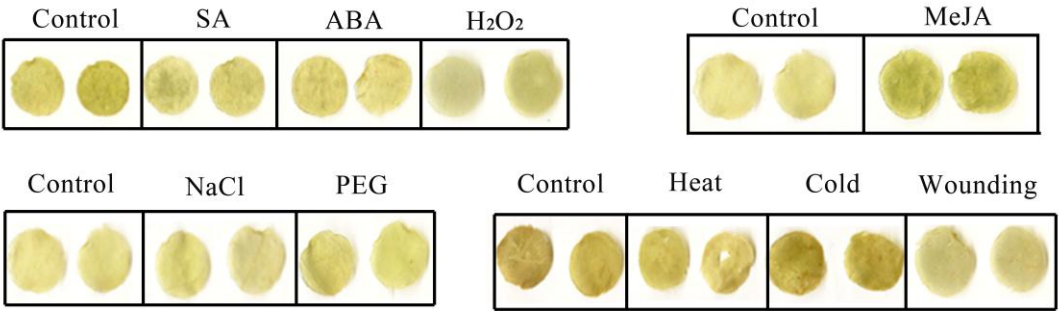

Supplement: Supplementary file 2 — GUS histochemical staining of the p121GUS tobacco leaves as negative control. (PDF 125 kb) [file 12870_2019_1678_MOESM2_ESM.pdf]

**Additional file 4:** PCR amplification of *CmLOX08* full length promoter.

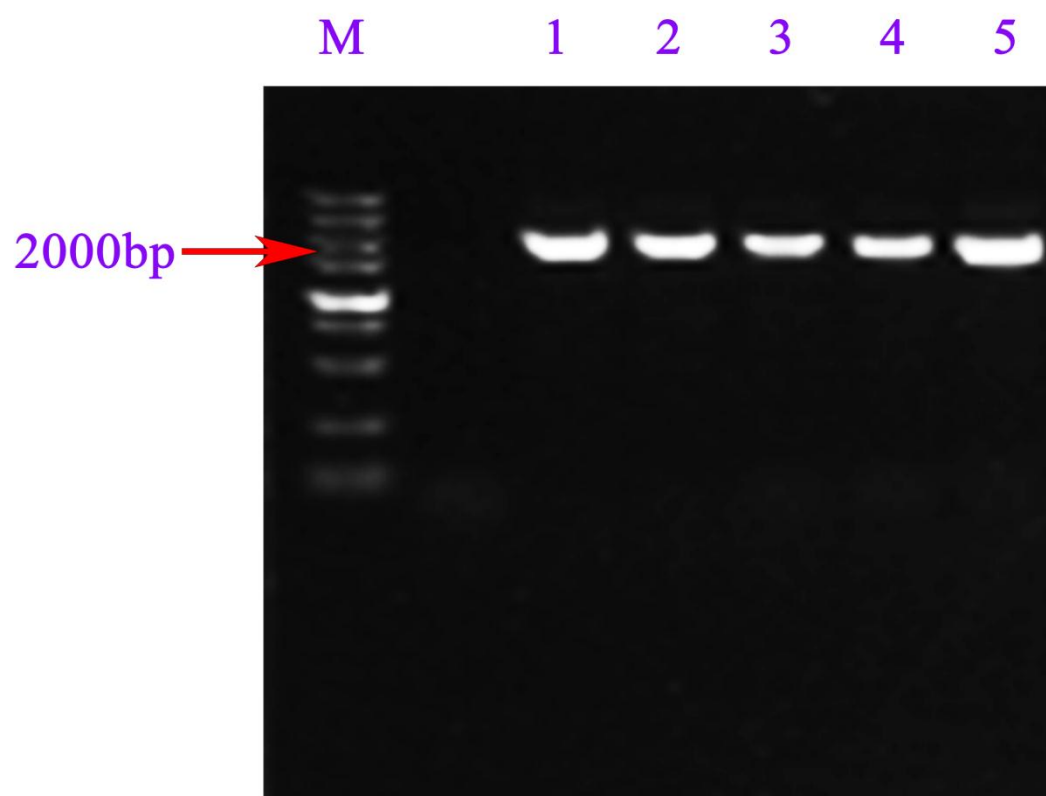

Supplement: Supplementary file 4 — PCR amplification of CmLOX08 full length promoter. Lane M: DL5000 DNA Marker; lane 1 to 5: CmLOX08 full length promoter fragment. (PDF 123 kb) [file 12870_2019_1678_MOESM4_ESM.pdf]

**Additional file 5:** The five different length recombinant vectors were verified by plasmid PCR.

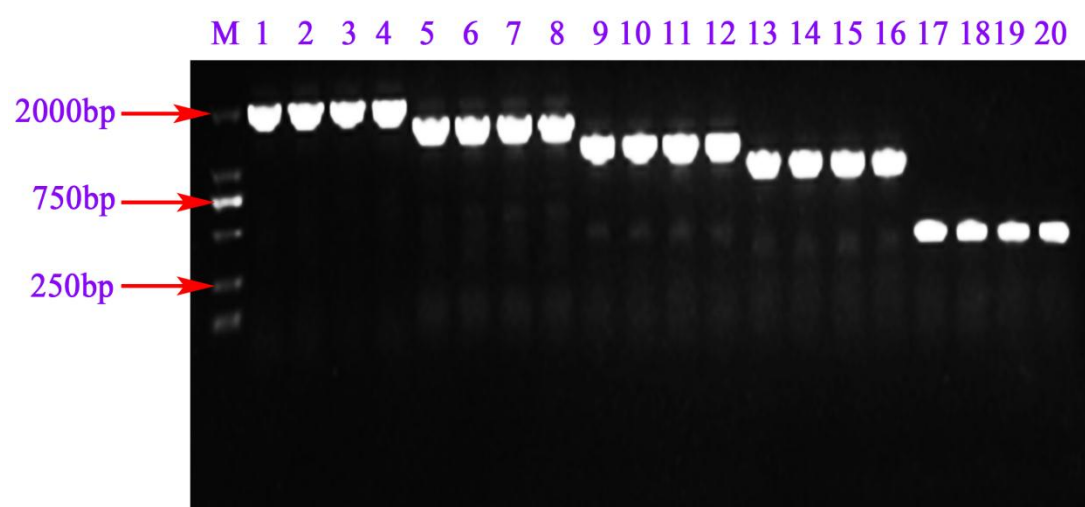

Supplement: Supplementary file 5 — The five different length recombinant vectors were verified by plasmid PCR. Lane M: DL5000 DNA Marker. Lane 1 to 4: LP1 (2054 bp); lane 5 to8: LP2 (1639 bp); lane 9 to 12:LP3 (1284 bp); lane 13 to 16: LP4 (1047 bp); lane 17 to 20: LP5 (418 bp). (PDF 126 kb) [file 12870_2019_1678_MOESM5_ESM.pdf]
