# Supplementary material for: Isolation and functional validation of the CmLOX08 promoter associated with signalling molecule and abiotic stress responses in oriental melon, Cucumis melo var. makuwa Makino
Source: BMC Plant Biol. 2019 Feb 15;19:75. doi: 10.1186/s12870-019-1678-1 (PMC6377772; doi:10.1186/s12870-019-1678-1)

Additional file 3: Expressions of *CmLOX08* at various time points under drought (a) and 50 mM NaCl (b) treatments.

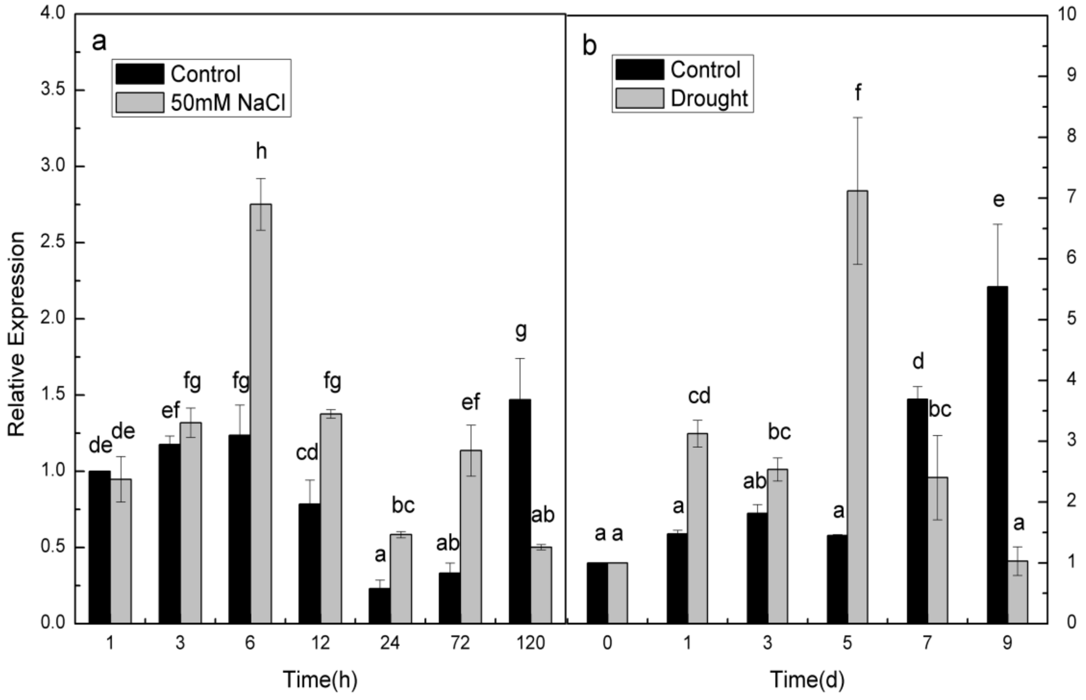

Supplement: Supplementary file 3 — Expressions of CmLOX08 at various time points under 50 mM NaCl (a) and drought (b) treatments. (PDF 148 kb) [file 12870_2019_1678_MOESM3_ESM.pdf]
